# Adaptive Fat Oxidation Is Coupled with Increased Lipid Storage in Adipose Tissue of Female Mice Fed High Dietary Fat and Sucrose

**DOI:** 10.3390/nu12082233

**Published:** 2020-07-27

**Authors:** Scott Fuller, Yongmei Yu, Timothy D. Allerton, Tamra Mendoza, David M. Ribnicky, Z. Elizabeth Floyd

**Affiliations:** 1 Pennington Biomedical Research Center, Louisiana State University System, Baton Rouge, LA 70808, USA; scott.fuller@louisiana.edu (S.F.); yongmei.yu@pbrc.edu (Y.Y.); timothy.allerton@pbrc.edu (T.D.A.); tamra.mendoza@pbrc.edu (T.M.); 2 Department of Kinesiology, University of Louisiana at Lafayette, Lafayette, LA 70506, USA; 3 Department of Plant Biology, Rutgers University, New Brunswick, NJ 08901, USA; ribnicky@sebs.rutgers.edu

**Keywords:** female, metabolism, botanical, Western diet, skeletal muscle, liver, adipose tissue, metabolic syndrome

## Abstract

Western diets high in fat and sucrose are associated with metabolic syndrome (MetS). Although the prevalence of MetS in women is comparable to that in men, metabolic adaptations in females to Western diet have not been reported in preclinical studies. This study investigates the effects of Western diet on risk factors for MetS in female mice. Based on our earlier studies in male mice, we hypothesized that dietary supplementation with extracts of *Artemisia dracunculus* L. (PMI5011) and *Momordica charantia* (bitter melon) could affect MetS risk factors in females. Eight-week-old female mice were fed a 10% kcal fat, 17% kcal sucrose diet (LFD); high-fat, high-sucrose diet (HFS; 45% kcal fat, 30% kcal sucrose); or HFS diet with PMI5011 or bitter melon for three months. Body weight and adiposity in all HFS groups were greater than the LFD. Total cholesterol level was elevated with the HFS diets along with LDL cholesterol, but triglycerides and free fatty acids were unchanged from the LFD. Over the three month period, female mice responded to the HFS diet by adaptive increases in fat oxidation energy in muscle and liver. This was coupled with increased fat storage in white and brown adipose tissue depots. These responses were enhanced with botanical supplementation and confer protection from ectopic lipid accumulation associated with MetS in female mice fed an HFS diet.

## 1. Introduction

The Western diet is characterized by high levels of saturated fat and sugars, along with meat and a low intake of fruits and vegetables [1]. This dietary pattern is strongly associated with obesity and the metabolic syndrome (MetS) [2], a cluster of risk factors associated with increased risk of developing type 2 diabetes and cardiovascular disease [3]. A generally agreed upon definition of MetS requires the presence of at least three of five risk factors: abdominal obesity or elevated waist circumference, insulin resistance/elevated blood glucose, elevated triglycerides, lowered high-density lipoprotein (HDL) cholesterol, and raised blood pressure [4,5]. By 2012, the prevalence of MetS in the United States reached 33% with a higher level in women than in men (35.6% versus 30.3%) [6]. The incidence of MetS is highest in lean or obese women whose dietary pattern consists of consuming high levels of fat and sweetened beverages [7], and the risk of developing type 2 diabetes increases more than ten-fold in women with MetS [8]. Considering the prevalence of MetS in women and the increased risk of developing MetS in women who consume a high-fat, high-sugar-content diet, preclinical studies examining the influence of dietary patterns on metabolic disorders in females are needed.

Preclinical studies of the impact of a high-fat, high-sucrose diet on metabolism carried out in male C57BL/6 mice show rapid changes in body weight, glucose tolerance, and peripheral insulin resistance [9,10]. However, there are few studies [11] examining the impact of the high-fat, high-sucrose dietary pattern in female rodents that could provide insight into the metabolic changes that lead to developing the risk factors of MetS in females. Moreover, women whose BMI is in the overweight-to-obese range report using dietary supplements for a variety of reasons that include improving or maintaining overall health or preventing health problems [12], but there is a paucity of preclinical data on the interaction of high-fat, high-sucrose dietary patterns with dietary supplements aimed at improving or maintaining metabolic health in females.

Our earlier preclinical studies in obese, insulin-resistant male mice show dietary supplementation with an ethanolic extract of *Artemisia dracunculus* L. (termed PMI5011) exerts positive effects on insulin signaling, lipid metabolism, and whole-body glucose homeostasis [13,14,15]. Further experiments extended these findings in males to demonstrate early dietary supplementation with PMI5011 protects against insulin resistance and ectopic lipid accumulation in the liver and skeletal muscle and that these effects are independent of any changes in adiposity or body mass [16]. Additional studies in male rodents showed that *Momordica charantia*, commonly known as bitter melon, acts as an insulin sensitizer by diminishing hepatic and skeletal muscle lipid accumulation and by reducing hepatic glucose production and lipogenesis [17,18,19,20]. Although these studies support the idea that botanical dietary supplements derived from sources such as *Artemisia dracunculus* L. and *Momordica charantia* can exert metabolically desirable effects in male mice exhibiting the risk factors for MetS [16,21], the beneficial effects may not be recapitulated in female mice exposed to a high-fat diet [22].

In the present study, we report that female mice challenged with a high-fat, high-sucrose diet developed impaired glucose tolerance, insulin resistance, and reduced metabolic flexibility in skeletal muscle with body weight gain. However, these changes occur in the context of a shift toward fatty acid oxidation in the skeletal muscle and liver, adipose tissue expansion via adipocyte hypertrophy, and accumulation of lipids in adipose tissue, including brown fat, but not skeletal muscle or liver, and maintenance of circulating triglycerides and non-esterified fatty acids at the levels observed in the low-fat-fed female mice. Thus, the females adapt to high dietary fat and sucrose by storing the lipids in adipose tissue and preferentially using lipids as a fuel source in skeletal muscle and liver. This corresponds to an absence of ectopic lipid storage in muscle or liver that is typically associated with MetS. These findings provide preclinical evidence that dietary intake of high levels of fat and sucrose in females is met with adaptive responses that protect against ectopic lipid accumulation. Moreover, botanical supplementation with PMI5011 or bitter melon partially mitigates the deleterious effects of a high-fat, high-sucrose diet in females.

## 2. Materials and Methods

### 2.1. Sourcing and Characterization of PMI5011 Extract

The PMI5011 botanical extract from *Artemisia dracunculus* L. was provided by the Botanical and Dietary Supplement Research Center at Pennington Biomedical Research Center. Detailed information about quality control, preparation, and biochemical characterization of PMI5011 has been previously reported [15,23,24,25,26,27,28]. Bitter melon was obtained from Verdure Sciences (Noblesville, IN, USA).

### 2.2. Experimental Animals

Four-week-old female C57BL/6J mice were obtained from Jackson Laboratories (Bar Harbor, ME). The mice remained reproductively intact throughout the four month study to increase relevance of the study to premenopausal women taking botanical dietary supplements. Previous studies show the estrous cycle stage does not significantly contribute to variability of molecular or metabolic outcomes measured in female mice [29]. All animal experiments were approved by the Pennington Biomedical Research Center Animal Care and Use Committee (protocol #1011). The animals were multiply housed with a 12 h light–dark cycle at 24 °C. Beginning at four weeks of age, the mice (*n* = 40) were fed a defined low-fat diet (LFD; 10% kcal fat, 17% kcal sucrose; Research Diets, #D12450H). At the end of one month, mice of similar body weight were randomly assigned to one of four groups (*n* = 10/group): continue on the defined LFD; begin high-fat, high-sucrose diet (HFS; 45% kcal fat, 30% kcal sucrose; Research Diets, custom diet #D08112601, Table S1); HFS with 1% (*w*/*w*) PMI5011; or HFS diet with 1% (*w*/*w*) bitter melon (custom diets formulated at Research Diets). The mice remained on these diets for twelve weeks. Body weight and food intake were measured weekly, and body composition was measured bi-weekly by nuclear magnetic resonance (Bruker, Billerica, MA, USA). Activity, food intake, and indirect calorimetry were measured at 12 weeks on each diet (TSE PhenoMaster, TSE Systems, Chesterfield, MO, USA). The mice were acclimated to the single-housed TSE chambers for 2 days prior to data collection over four days. At the end of the study, the mice were euthanized between 7 and 11 a.m. after fasting for 4–5 h. Because mice eat very little at the end of the dark cycle (Figure S1), the mice were considered to be in a postabsorptive state when tissues were collected.

### 2.3. Glucose and Insulin Tolerance Tests, Blood Chemistry, and Hepatic Triglyceride Content

For glucose tolerance testing (GTT), the amount of glucose administered was normalized to body weight at 2.5 mg gm glucose/kg body weight. For insulin tolerance testing, 0.02 units of insulin/mouse (HumulinR) was administered. The female mice were fasted 4 h prior to administering the glucose or insulin solution by intraperitoneal injection. Fasting glucose levels were measured in whole blood using a Breeze2 glucometer (Bayer, Leverkusen, Germany). Serum non-esterified fatty acids (Abcam, Cambridge, MA, USA), triglyceride (Eagle Diagnostics, Cedar Hill, TX, USA), and cholesterol (Cell Biolabs, San Diego, CA, USA) levels were assayed according to the manufacturers’ instructions. Serum ALT, AST, total bilirubin and albumin were assayed in the Pennington Biomedical Clinical Core. Hepatic triglycerides were assayed as previously described [16].

### 2.4. Immunohistochemistry

A portion of mixed gastrocnemius muscle, liver, gonadal (gWAT) and inguinal (iWAT) white adipose tissue, and brown adipose tissue (BAT) was fixed in 10% formalin, embedded in paraffin, and sectioned onto slides. Visible fat was carefully removed from the skeletal muscle and liver tissue prior to tissue fixation. The sections were hematoxylin and eosin (H&E) stained and scanned (NanoZoomer Digital Pathology, Hamamatsu Corp., Bridgewater, NJ, USA).

### 2.5. Fatty Acid Oxidation Assay

Mixed gastrocnemius muscle homogenates were prepared as described [22]. Palmitate oxidation was assessed in the whole-muscle homogenates as described by Hulver et al. [30] with ^14^CO_2_ collected over 60 min. When present, pyruvate was added at a final concentration of 1 mM. CO_2_ levels were normalized to total protein and palmitate oxidation reported as nmol CO_2_/mg protein/hour.

### 2.6. Analysis of Protein Expression

Skeletal muscle and liver lysates were prepared from powdered tissue by homogenizing in 25 mM HEPES, pH 7.4, 1% Igepal CA630, 137 mM NaCl, 1 mM PMSF, 10 μg/mL aprotinin, 1 μg/mL pepstatin, 5 μg/mL leupeptin, 10 mM Na_4_P_2_O_7_, 100 mM NaF, and 2 mM NaVO_4_ using a Sonifier 450 homogenizer (VWR, Radnor, PA, USA). The samples were centrifuged at 12,000× *g* for 10 min at 4 °C. Protein concentrations were determined using a BCA assay (Thermo Fisher Scientific, Rockford, IL, USA) according to the manufacturer’s instructions. Tissue supernatants (50 μg) were resolved by SDS-PAGE and subjected to immunoblotting using chemiluminescence detection (Thermo Fisher Scientific, Rockford, IL, USA) and quantified using Image J software after normalizing to β-actin or when appropriate, total protein kinase B (AKT) or total AMP kinase alpha subunit AMPKα. Nitrocellulose membranes were incubated with antibodies for 1–2 h at room temperature or overnight at 4 °C as indicated (Table S2).

### 2.7. Analysis of Gene Expression

Total RNA was purified from powdered skeletal muscle tissue or liver using Direct-zol RNA MiniPrep (ZYMO Research, Irvine, CA, USA). In each case, RNA (500 ng) was reverse transcribed using MultiScribe Reverse Transcriptase (Applied Biosystems, Thermo Fisher Scientific, Waltham, MA, USA) with random primers at 37 °C for 2 h. Real-time PCR was performed with PowerUP SYBR Green Master Mix (Applied Biosystems) according to the manufacturer’s instructions, using the 7900 Real-Time PCR system and universal cycling conditions (50 °C for 2 min; 95 °C for 10 min; 40 cycles of 95 °C for 15 s and 60 °C for 1 min; followed by 95 °C for 15 s, 60 °C for 15 s, and 95 °C for 15 s). The assays were performed in triplicate, and the results were normalized to Cyclophilin B mRNA and analyzed using the 2^−ΔΔCT^ method with the control diet used as the calibrator. A gene list and primers are provided in Table S3.

### 2.8. Ex Vivo Lipolysis Assay

Adipose explants (gWAT) were washed in phosphate-buffered saline PBS and dissected into individual 20–30 mg pieces. Different explants from the same mice were used in the lipolysis experiments. Explants were incubated for 2 h in phenol red-free DMEM (low glucose) media at 37 °C for induction of basal lipolysis or in the presence of isoproterenol (1 nM) to induce beta-adrenergic stimulated lipolysis. Explants were transferred to wells with fresh media containing 10 nM insulin or insulin-free media for 30 min to suppress lipolysis. Lipolysis was quantified by measuring glycerol in the media by a colorimetric assay (Sigma Aldrich) and normalized to tissue explant weight (gm).

### 2.9. Statistical Analysis

**Figure 1:**
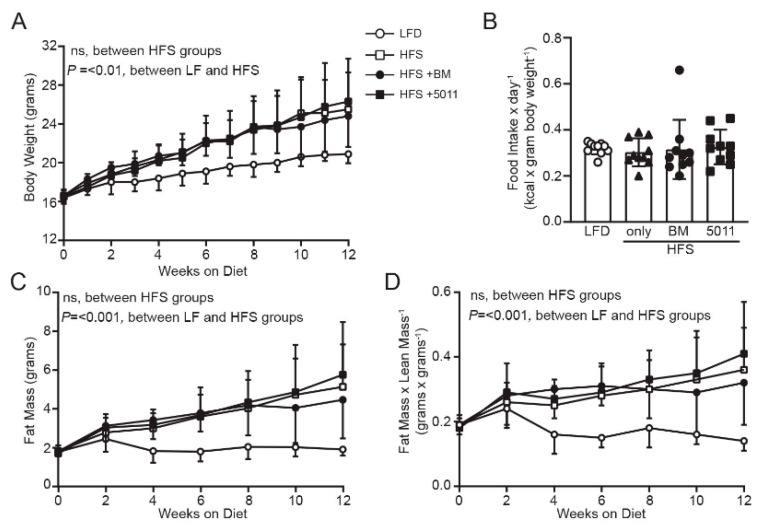
High-fat, high-sucrose (HFS) diet results in weight gain and increased adiposity independent of food intake or dietary supplementation with botanical extracts. Body weight (**A**) increased in the HFS groups versus low-fat (LF)-fed controls but did not differ between HFS groups. Food intake (**B**) did not differ between groups. Fat mass (**C**) was elevated in all HFS groups compared to LF-fed mice, but did not differ between the HFS-fed groups. Fat mass/lean mass ratio (**D**) was decreased in the LF group compared to that in all HFS groups, which did not differ from each other. (*n* = 10 per group), statistical significance was set at *p* < 0.05. Variability is expressed as mean ± SD.

**Figure 2:**
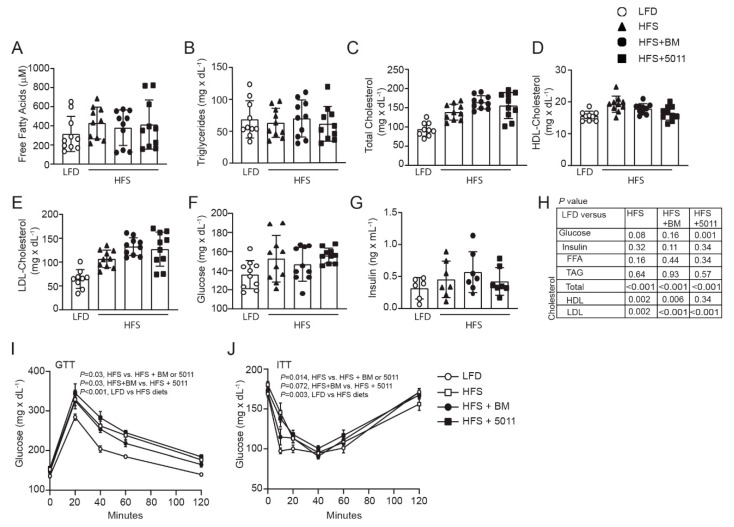
Effects of HFS diets on serum lipids and glucose homeostasis in the female mice. Serum free fatty acids (**A**) and triglycerides (**B**) were not affected by the HFS diets although total (**C**) and low-density lipoprotein (LDL) (**E**), but not high-density lipoprotein (HDL) (**D**) cholesterol were increased with the HFS diets. Fasting glucose (**F**), but not insulin (**G**) was increased by the HFS diet. Glucose levels were unchanged from the low-fat-diet (LFD) group with botanical supplementation (**F**), but glucose tolerance (**I**) and insulin sensitivity (**J**) was impaired in all HFS-fed groups. Statistical significance (**H**) for each panel was set at *p* < 0.05, (*n* = 10 per group), variability is expressed as mean ± SD.

**Figure 3:**
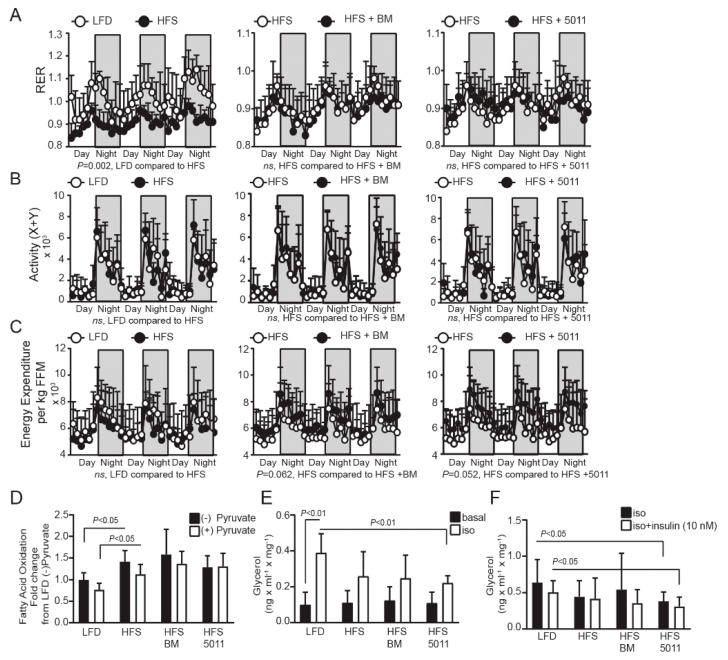
Effects of HFS diet and botanical supplementation on substrate utilization in vivo and ex vivo in skeletal muscle and adipose tissue. The respiratory exchange ratio (RER) (**A**) reflects a shift toward fatty acid oxidation with HFS diets that was not altered by botanical supplementation. Activity (**B**) and energy expenditure (**C**) were not altered by diet. Fatty acid oxidation (FAO) was measured ex vivo in skeletal muscle (**D**) in the presence and absence of pyruvate to assess the impact of diet on substrate switching and metabolic flexibility in skeletal muscle. Adipose tissue explants were exposed to adrenergic stimulation by isoproterenol (**E**) and lipolytic rates were measured. Isoproterenol-induced lipolysis was reduced in the HFS compared to that in the LF controls, and the blunted response was accentuated by PMI5011 supplementation. Adipose tissue explants were treated with isoproterenol + or – insulin (**F**) to determine insulin suppression of lipolytic rate. HFS-fed mice supplemented with PMI5011 showed enhanced insulin-mediated suppression of lipolysis. Statistical significance was set at *p* < 0.05, (*n* = 10 per group), variability is expressed as mean ± SD.

Normal distribution of the data for glucose and insulin levels, food intake, and body weight was determined using the D’Agostino–Pearson omnibus K2 normality test. Western blot data were quantified using ImageJ software. Statistical significance was determined using an unpaired two-tailed t test or 2-way ANOVA when diet and time were independent factors (Figure 1A,C,D; Figure 2I–J; and Figure 3A–C). All statistical analyses were carried out using JMP Pro14 (SAS Institute) and GraphPad Prism 8 software (GraphPad Software, La Jolla, CA, USA). Variability is expressed as the mean ± standard deviation.

## 3. Results

### 3.1. HFS Diet Induces Body Weight and Fat Mass Gain in Females That Is Not Reduced by Botanical Supplementation

Figure 1A depicts body weight assessed weekly throughout the duration of the study. The mice maintained on the defined low-fat diet exhibited consistently lower body weight throughout the study period, while the body weights of the groups fed an HFS diet did not significantly differ from one another. Food intake normalized to body weight did not differ between groups over the duration of the study (Figure 1B). Fat mass as determined by NMR was higher in all groups fed HFS diet compared to that of mice fed the LF diet (Figure 1C). Analysis of the fat mass to lean mass ratio support body weight gain due to increased fat mass in the HFS-fed mice (Figure 1D). Collectively, these results indicate that deleterious effects on body composition associated with the HFS diet in female mice were not significantly attenuated by PMI5011 or bitter melon dietary supplementation.

### 3.2. Females Fed an HFS Diet Develop Glucose Intolerance and Insulin Resistance That Is Not Reversed by Botanical Supplementation

Although non-esterified fatty acids (Figure 2A) and triglycerides (Figure 2B) were unaffected by the HFS diets in the females, total and LDL-cholesterol levels increased with the HFS diets (Figure 2C–E,H). This corresponded to elevated blood glucose (Figure 2F), but not insulin (Figure 2G) with the HFS diet alone that was reflected in impaired glucose tolerance in the HFS-fed females compared to the LFD, with the most negative impact on glucose tolerance shown in the HFS + PMI5011 group (Figure 2I). Statistically significant differences were found in blood glucose and glucose tolerance with botanical supplementation when compared to the HFS diet alone that did not translate into glucose tolerance comparable to the low-fat-diet-fed females. Insulin tolerance testing (ITT) also indicated that the LFD-fed mice showed greater responsiveness to insulin than any of the HFS groups (Figure 2J).

### 3.3. HFS Diet Increases Fatty Acid Oxidation as a Fuel Source but Reduces Skeletal Muscle Metabolic Flexibility—Botanical Supplementation Does Not Improve Substrate Switching Ability

Metabolic flexibility is an indicator of metabolic health originally defined as “the capacity to switch from predominantly lipid oxidation and high rates of fatty acid uptake during fasting conditions to the suppression of lipid oxidation and increased glucose uptake, oxidation, and storage under insulin-stimulated conditions” [31]. Metabolic flexibility more broadly refers to physiological adaptability in adjusting fuel oxidation to nutrient availability and is affected by diurnal patterns of food intake or exercise [32,33]. Although mice do not voluntarily fast, and the respiratory exchange ratio (RER) over a three day period parallels the food quotient (FQ) determined by macromolecular composition of diets [34] (Figure S1); the respiratory exchange ratio (RER) during the daily cycles reflects adaptation to nutrient intake and offers insight into overall metabolic flexibility. Comparisons of the RER between LF-fed mice and HFS-fed groups indicate that HFS feeding shifts fuel selection toward fat oxidation under fasting conditions during the light cycle, but without a shift toward predominantly carbohydrate metabolism during the dark cycle (as seen in the low-fat diet, LFD) (Figure 3A), even though sucrose levels are elevated compared to the LFD. The preference for fatty acid oxidation was not altered by bitter melon or PMI5011 supplementation in the HFS-fed female mice (Figure 3A). Fuel preferences were also unrelated to activity (Figure 3B) or energy expenditure (Figure 3C) among the groups during the daily cycles. To determine whether the metabolic inflexibility suggested by the RER was reflected in a preference to oxidize fatty acids in the skeletal muscle of HFS-fed female mice, we assayed fatty acid oxidation in vitro using mixed-fiber-type gastrocnemius muscle homogenates. The transition between fasting and feeding was simulated by adding pyruvate as a competing glucose-derived carbon source to mimic the fed state (Figure 3D). An impairment in the ability to robustly switch between fat and carbohydrate oxidation when transitioning from the fasted to the fed state is a hallmark characteristic of MetS and is indicated by a failure to suppress fatty acid oxidation in skeletal muscle homogenates in the presence of pyruvate [35]. While fatty acid oxidation was increased by the HFS diet, fatty acid oxidation was not suppressed by pyruvate, suggesting the elevated dietary intake of fat and sucrose induced metabolic inflexibility in skeletal muscle of the females. Unlike our previously reported findings in females fed a high fat diet [22], botanical supplementation in conjunction with an HFS diet did not shift skeletal muscle toward carbohydrate oxidation when challenged with pyruvate.

### 3.4. Effect of the HFS Diet on Ex Vivo Measures of Lipolysis in Adipose Tissue

Systemic metabolic flexibility is also determined by the release of fatty acids from white adipose tissue during fasting to support fatty acid uptake as a fuel source in skeletal muscle and insulin-mediated suppression of lipolysis under feeding conditions when carbohydrates are the primary fuel source [33]. Figure 3C depicts lipolysis in basal conditions and in response to adrenergic stimulation of lipolysis with isoproterenol in visceral adipose tissue collected from fasting female mice. The LFD-fed control group shows a robust, approximately fourfold increase in lipolysis when treated with isoproterenol. Adrenergic-mediated lipolysis in the HFS alone and HFS with bitter melon groups did not differ significantly from that in the LF-fed group, but stimulation of lipolysis was significantly blunted in the HFS group supplemented with PMI5011 when compared to the LF-fed group. In addition, the HFS group supplemented with PMI5011 enhanced the ability of insulin to suppress lipolysis relative to the LFD-fed group (Figure 3D). However, the visceral adipose tissue in each of the groups did not show insulin-mediated suppression of lipolysis relative to the isoproterenol-treated lipolysis within the group.

### 3.5. Effects of HFS Diet on Markers of Insulin Sensitivity and Fatty Acid Oxidation in Skeletal Muscle

**Figure 4:**
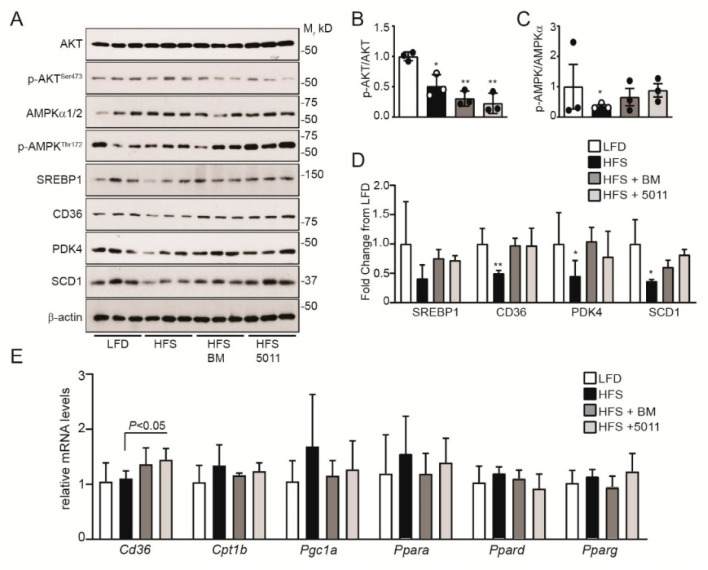
HFS diet-mediated changes in protein markers of lipid metabolism are altered by botanical supplementation in skeletal muscle. Protein and gene expression were assayed in skeletal muscle by Western blotting (**A**) and RT-PCR (**E**). Western blot density quantification is depicted for protein kinase B (AKT) activation (**B**), AMPKα activation (**C**) and fold change of lipid metabolism protein markers (**D**). RT-PCR analysis of mRNA expression (**E**) indicates that transcription was not affected by diet or botanical supplementation, although *cd36* mRNA expression differed between the HFS group and the PMI5011-supplemented mice. Statistical significance was set at * *p* < 0.05, ** *p* < 0.01 compared to LFD (**B**–**D**); variability is expressed as mean ± SD. *n* = 3 (**A**–**D**); *n* = 5 (**E**).

Skeletal muscle is the primary site of insulin-stimulated glucose uptake and metabolism [36]. In male mice fed an HFS diet, insulin-stimulated glucose uptake is reduced in the skeletal muscle, in agreement with decreased RER [10]. In the females fed an HFS diet, insulin signaling was significantly suppressed when assayed by protein kinase B (AKT) activation in response to acute insulin stimulation (Figure 4A,B). Supplementation with PMI5011 or bitter melon led to more pronounced inhibition of AKT phosphorylation when compared to the HFS diet alone (Figure 4A,B). In contrast, markers of fatty acid oxidation were upregulated by botanical supplementation of the HFS diet. Protein expression of the scavenger receptor CD36 (Figure 4A) that facilitates long-chain fatty acid uptake in skeletal muscle [37] was upregulated by botanical supplementation in the HFS diet, but not by the HFS diet alone. Upregulation of CD36 in HFS with botanical supplementation was accompanied by increased activation of the AMP-kinase alpha subunits (Figure 4A,C), consistent with increased fatty acid oxidation in the skeletal muscle of the females. Protein expression of pyruvate dehydrogenase kinase-4 (PDK4), a sensitive indicator of fatty acid oxidation [38,39], was also upregulated with botanical supplementation of the HFS diet compared to the HFS diet alone (Figure 4A,D). Consistent with HFS diet-stimulated increased fatty acid oxidation in the skeletal muscle, protein markers of lipid synthesis (sterol regulatory element-binding protein-1/SREBP1 and stearoyl-CoA desaturase-1/SCD1) were unchanged or downregulated in the skeletal muscle of the HFS-fed females, independent of botanical supplementation (Figure 4A,D).

While increased CD36 protein expression corresponded to transcriptional regulation of *Cd36* mRNA by the botanicals in the HFS diet (Figure 4E), HFS diet alone or with botanical supplementation did not transcriptionally regulate the gene expression of markers that drive fatty acid oxidation (*Cpt1b, Pgc1a, Ppara, Ppard*) in the skeletal muscle of the females.

### 3.6. Effects of HFS Diet on Markers of Fatty Acid Oxidation and Insulin Sensitivity in the Liver

**Figure 5:**
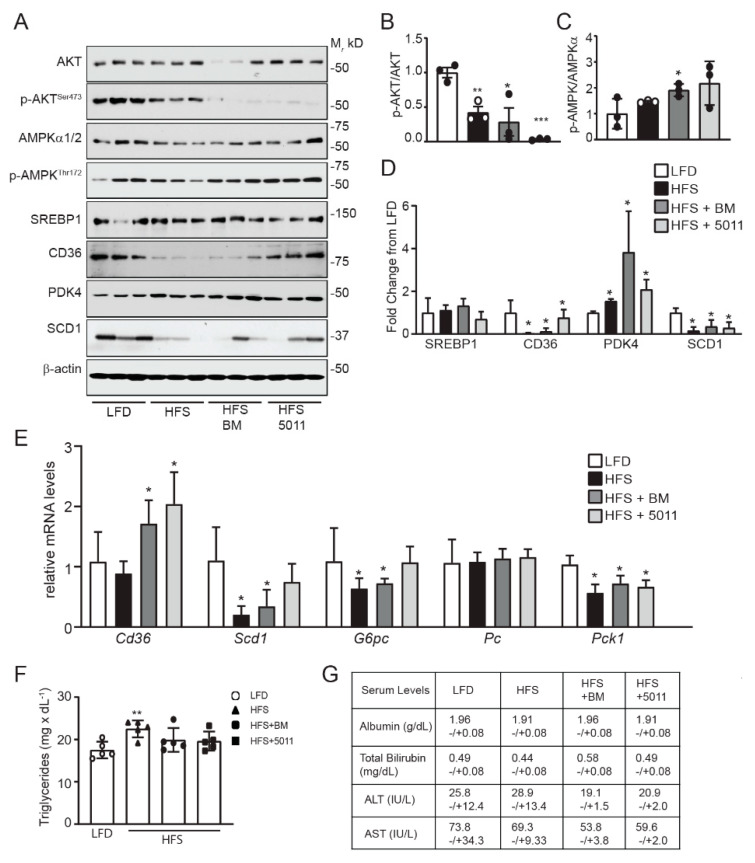
Hepatic markers of insulin responsiveness and lipid metabolism are altered by the HFS diets in female mice. Protein and gene expression were assayed in liver by Western blotting (**A**) and RT-PCR (**E**). Western blot density quantification is depicted for AKT activation (**B**), AMPKα activation (**C**) and fold change of lipid metabolism protein markers (**D**). Western blot analysis indicates that insulin responsiveness was diminished in livers of HFS-fed mice, with botanical supplementation intensifying this effect (**B**), as indicated by reduced AKT phosphorylation. Panel (**C**) shows increased activation of AMPK with botanical supplementation, consistent with increased fatty acid utilization. Expression of CD36, pyruvate dehydrogenase kinase-4 (PDK4), and SCD-1 proteins (**D**) was altered by each HFS diet compared to the LFD. Hepatic mRNA expression of *cd36*, *scd-1*, and *pck-1* was significantly altered by the HFS diets (**E**). Hepatic triglyceride levels were increased by the HFS diet but not in the HFS diet supplemented with bitter melon or PMI5011 (**E**). Liver function was not affected by the HFS in the females (**G**). Statistical significance was set at * *p* < 0.05, ** *p* < 0.01, *** *p* < 0.001 compared to LFD (**B**–**E**); variability is expressed as mean ± SD. *n* = 3 (**A**–**D**); *n* = 5 (**E**–**G**).

Skeletal muscle, adipose tissue, and the liver are the major sites controlling fatty acid metabolism and adaptation of lipid metabolism to nutrients. In male mice fed an HFS diet, triglyceride accumulated in the liver coincides with a plateau in adipose tissue expansion [10,40], indicating the ectopic lipids released by lipolysis were stored as lipids in the liver rather than oxidized as a hepatic fuel source. In the current study, the HFS diet corresponded to substantial downregulation of hepatic insulin responsiveness (Figure 5A,B) that was further reduced with botanical supplementation. While markers of lipid synthesis were unchanged or downregulated at the protein level (Figure 5A,D; SREBP1 and SCD1), we found that CD36 protein was significantly reduced by the HFS diet but returned to the levels observed in the LFD with PMI5011 supplementation. *Cd36* mRNA levels increased with botanical supplementation of the HFS diet, but not with the HFS diet alone, suggesting the botanicals stimulated compensatory upregulation of *Cd36* in response to the low CD36 protein levels (Figure 5E). Bitter melon HFS supplementation also restored *Scd1* mRNA expression to LFD-fed levels. The pattern of hepatic *Scd1* mRNA is particularly noteworthy, in that, studies of diet-induced obesity and metabolic dysfunction in male mice typically show increased expression of this lipogenic gene in response to high-fat or high-sucrose feeding [41,42]. Like upregulation of *Cd36* mRNA, botanical-mediated increased *Scd1* mRNA levels in the liver of the female mice may represent compensatory upregulation. However, AMPK activation (Figure 5A,C) and PDK4 protein (Figure 5A,D) are markers of fatty acid oxidation that were upregulated by the HFS diet or the HFS diet supplemented with PMI5011 or bitter melon. Inhibition of insulin responsiveness by the HFS diets was also associated with downregulation of *Pck1*, *G6pc*, and *Pc* mRNA encoding proteins that regulate gluconeogenesis (Figure 5E). Taken together, these data indicate hepatic fatty acid oxidation is stimulated in the HFS-fed females in the presence of insulin resistance. Nonetheless, there was a small (1.3-fold), but significant, increase in hepatic triglyceride content of the HFS-fed mice compared to the LFD-fed mice, but this increase was blunted by supplementation with bitter melon or PMI5011 (Figure 5F). Finally, there was no decline in liver function in the female mice fed the HFS diets (Figure 5G).

### 3.7. Effect of HFS Diet in Females on Lipid Accumulation in Skeletal Muscle, Liver, and Adipose Tissue

**Figure 6:**
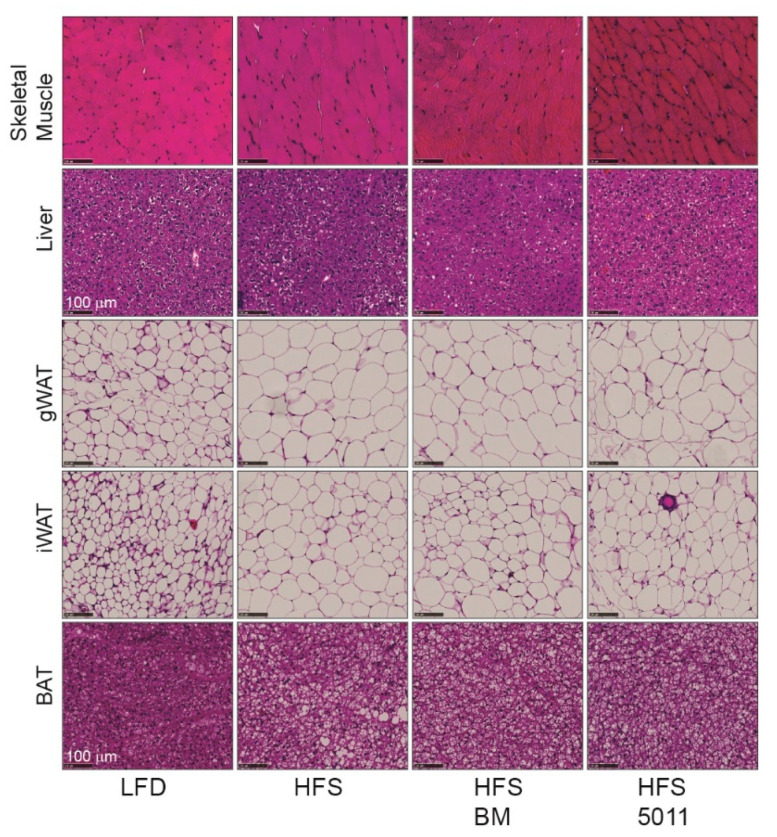
Tissue morphology of skeletal muscle, liver, white adipose, and brown adipose tissues shown by hematoxylin and eosin staining. Lipid accumulation in skeletal muscle was not higher in the HFS diets compared to the LFD. A small increase in hepatic lipid content with the HFS was attenuated by botanical supplementation. Lipid accumulation in perigonadal (gWAT) and inguinal (iWAT) white adipose tissue was increased by adipocyte hypertrophy in all HFS-fed groups. Brown-fat lipid accumulation occurred in unilocular and multilocular lipid droplets in the females fed the HFS diet but was unilocular and multilocular droplets in the HFS alone diet and predominantly multilocular lipid droplets in the botanical supplemented HFS-fed diets. The images are representative of images from four mice/group. The black bar length represents 100 µm.

Interestingly, the HFS diets did not correspond to morphological changes indicating increased lipid accumulation in the skeletal muscle (Figure 6). The morphology of the liver as evaluated by H&E staining reflected the small increase in hepatic triglyceride accumulation in the HFS-fed females compared to the LFD-fed mice that did not occur with the botanical supplementation. Strikingly, expansion of the white perigonadal and inguinal adipose tissue by adipocyte hypertrophy in the HFS-fed females was apparent. There was also increased lipid storage in the brown fat that occurred in unilocular and multilocular lipid droplets in the HFS diet alone, but predominantly multilocular lipid droplets when the HFS diet was supplemented with either botanical extract.

## 4. Discussion

High levels of dietary fats and refined carbohydrates are commonly consumed as part of the current Western diet. This dietary pattern is associated with developing MetS, a set of metabolic changes that predict an increased risk of developing type 2 diabetes [43]. As consumption of the Western diet has continued, the incidence of MetS has increased in lean or obese women, posing a significant health issue for women. Even so, there are few preclinical studies examining the impact of the high-fat, high-sugar diet on metabolic changes in females.

In the current study, we examined the metabolic changes associated with dietary intake of a high-fat, high-sucrose diet over three months in fertile female mice. In contrast to male mice fed a high-fat, high-sucrose diet [10], the female mice exhibited a set of metabolic adaptations that combined to reduce the impact of lipid overload in the skeletal muscle and liver while promoting storage of excess energy as lipids in the adipose tissue. Maintenance of plasma non-esterified fatty acid and triglyceride levels comparable to the levels observed in the low-fat-fed females along with reduced RER, a shift toward fatty acid oxidation in the skeletal muscle, increased markers of fatty acid uptake and oxidation in the skeletal muscle and liver, and reduced insulin signaling in the skeletal muscle and liver suggests the females responded to the lipid overload by shifting fuel utilization to fatty acid oxidation in the muscle and liver. Restoration of CD36 protein expression toward LFD levels in tandem with increased AMPKα subunit activation in the botanically supplemented groups indicates that these botanicals could play a role in enhancing fatty acid uptake and oxidation in skeletal muscle and liver, thereby inhibiting ectopic lipid accumulation in the female mice. At the same time, insulin action in the skeletal muscle and liver was inhibited and corresponded to reduced expression of markers of gluconeogenesis, indicating an integrated response to the HFS diet in the females resulting in a preference for lipids as a fuel source. Although high sucrose consumption is characteristically associated with hepatic lipogenesis and triglyceride accumulation due to the high fructose content [44], the absence of ectopic lipid accumulation in the skeletal muscle and liver in the HFS-fed females indicates these adaptive mechanisms combine to shield the skeletal muscle and liver from the detrimental effects of ectopic lipid accumulation observed with MetS. This is particularly relevant to human consumption of sugar-sweetened foods and beverages given the evidence that fructose derived from sucrose, rather than glucose appears to be responsible for the increase in serum triglycerides and ectopic lipid accumulation observed in MetS [45].

Maintenance of blood glucose levels indicates the shift toward lipid oxidation did not come at the expense of glucose overload with the high-sucrose dietary intake. Although not examined in our studies, glucose storage as glycogen in the skeletal muscle is a possible mechanism for sequestering the excess glucose. However, insulin signaling inhibition in the skeletal muscle and liver indicates a state of insulin resistance in the muscle and liver, reducing the likelihood of insulin-mediated stimulation of glycogen storage in either tissue. Nonetheless, there is evidence that insulin-independent pathways to glycogen production that rely on AMPK activation operate in skeletal muscle [46], a change that occurred in both skeletal muscle and the liver of the females. Expansion of the white adipose tissue in response to the high-fat, high-sucrose diets indicates that the metabolic adaptations are a combination of increased lipid oxidation combined with increased storage of neutral lipids in the adipose tissue. This is consistent with our finding that relative to the low-fat-fed females, lipolysis is suppressed in the perigonadal fat of the females fed the high levels of fat and sucrose, an outcome predicted by the increased hepatic fatty acid oxidation [47]. The ability of the females to store excess macronutrients as neutral lipids is emphasized by the brown-fat accumulation of multilocular lipid droplets. These multilocular droplets are typically found in brown fat, while the appearance of unilocular lipid droplets is associated with lipid storage characteristic of white fat.

The role of estrogen was not tested in our study, but there is abundant evidence that estrogens stimulate a range of transcriptional and post-translational changes that stimulate fatty acid utilization in the liver and skeletal muscle [48,49,50,51] and promote lipid storage in subcutaneous but not visceral fat in women [52]. The impact of estrogen on metabolism in females likely contributes to the relative protection from ectopic lipid accumulation found in MetS, but the adaptations to the HFS diet observed in our study occurred over a relatively short exposure when considered as a model of the chronic dietary intake of the Western diet in women. The metabolic adaptations observed in the twelve-week exposure to the HFS diet did not protect the females from becoming glucose intolerant, an indicator of insulin resistance that constitutes the underlying risk factor in MetS. Thus, the metabolic adaptations we observed in the females may not persist as the adipose tissue reaches a plateau for expansion with longer exposure to the HFS diet.

Given the rising prevalence of MetS in women and data indicating that women use dietary supplements at higher rates than men [53,54,55], we asked whether botanical supplements reported to improve glucose homeostasis and reduce ectopic lipid accumulation [14,16,25,56,57] would modulate the metabolic effects of the HFS diet in females. We previously reported that female mice respond in a generally less advantageous manner to botanically derived nutritional supplements than males in the context of a high-fat diet [22]. However, botanical supplementation in this cohort of female mice evoked a set of metabolically beneficial responses compared to HFS diet alone that were independent of changes in body weight. Suppression of insulin signaling by PMI5011 and bitter melon in the skeletal muscle and liver with the HFS diet was surprising given previous studies showing the botanicals enhance insulin signaling in the setting of high fat diet–induced insulin resistance [15,25,56,57]. However, botanical-mediated reduced insulin signaling was coupled with increased expression of markers of fatty acid oxidation in the skeletal muscle and liver and, in the case of PMI5011, suppression of fatty acid release from the visceral adipose tissue. Unlike exercise-induced reductions in adipose tissue mass [58], bitter melon and PMI5011 supplementation supported white adipose tissue expansion and promoted accumulation of multilocular lipid droplets in the brown fat that provide a source of lipids for the high level of fatty acid oxidation in brown fat [59]. Thus, botanical supplementation augmented the metabolic adaptations to HFS dietary intake in the females that combine to protect skeletal muscle and liver from ectopic fat accumulation by promoting lipid oxidation as a fuel source while maintaining the capacity of adipose tissue to store excess lipids.

## Supplementary Materials

The following are available online at https://www.mdpi.com/2072-6643/12/8/2233/s1, Figure S1: Correlation between RER and food quotient (FQ). Table S1: Custom HFS diet composition. Table S2: Antibody information. Table S3: Gene name and primer information
