# Supplementary material for: Adaptive Fat Oxidation Is Coupled with Increased Lipid Storage in Adipose Tissue of Female Mice Fed High Dietary Fat and Sucrose
Source: Nutrients. 2020 Jul 27;12(8):2233. doi: 10.3390/nu12082233 (PMC7469071; doi:10.3390/nu12082233)
Supplement: Supplementary file 1 [file nutrients-12-02233-s001.zip › Fuller et al_Supplementary Figure S1_v072620.pdf]

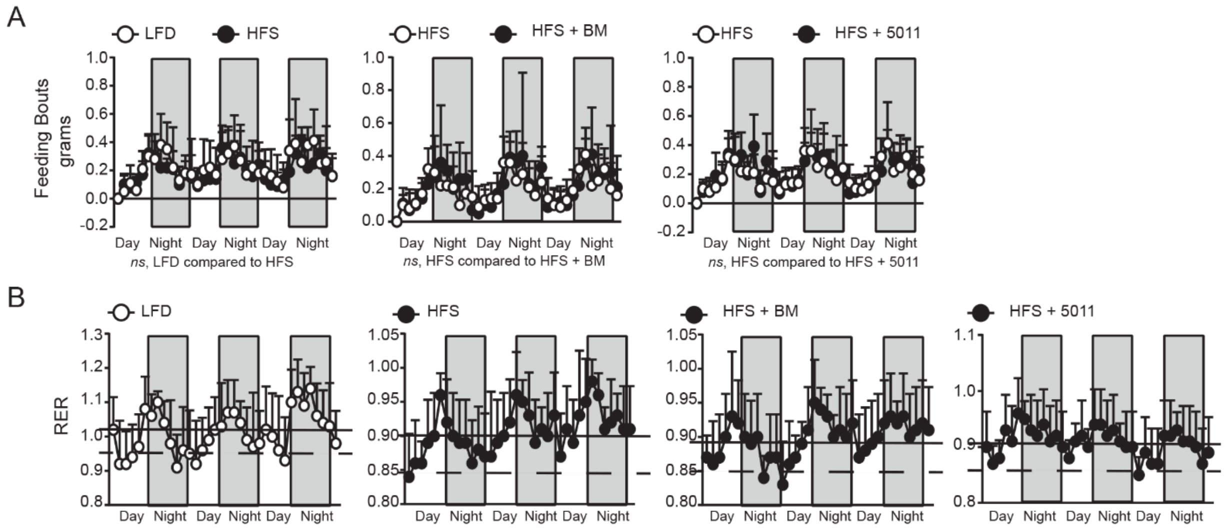

Figure S1. Correlation between RER and food quotient (FQ). Peak food intake occurs early in the dark phase (A) and correponds to changes in RER (B). The average RER (B) is shown as a horizontal solid line and average food quotient (B) is shown as a horizontal dashed line. Average RER is 1.01 -/+ 0.062 (LFD); 0.907 -/+ 0.033 (HFS); 0.898 -/+ 0.028 (HFS + BM) and 0.908 -/+ 0.025 (HFS + 5011). The FQ was 0.925 (LFD); 0.822 (HFS); 0.822 (HFS + BM) and 0.822 (HFS +5011).
