# Supplementary material for: Adaptive Fat Oxidation Is Coupled with Increased Lipid Storage in Adipose Tissue of Female Mice Fed High Dietary Fat and Sucrose
Source: Nutrients. 2020 Jul 27;12(8):2233. doi: 10.3390/nu12082233 (PMC7469071; doi:10.3390/nu12082233)
Supplement: Supplementary file 1 [file nutrients-12-02233-s001.zip › Fuller et al_Table S1_Custom diet composition.pdf]

A.

LFD: https://researchdiets.com/formulas/d12450H

B. HFS Custom Diet: D08112601

**Diet Composition Macromolecules**	**High Fat, High Sucrose Diet Custom/Research Diets #D08112601**	
**%**	**grams**	kcal
Protein	24	20
Carbohydrate	41	35
Fat	24	45
**Total**		100
**Ingredients**		
Casein, 80 Mesh	200	800
L-Cystine	3	12
Corn Starch	0	0
Maltodextrin 10	50	200
Sucrose	295.6	1182
Fructose	0	0
Cellulose	50	0
Soybean Oil	25	225
Lard	177.5	1598
**Total**	858.15	4057
