# Supplementary material for: Adaptive Fat Oxidation Is Coupled with Increased Lipid Storage in Adipose Tissue of Female Mice Fed High Dietary Fat and Sucrose
Source: Nutrients. 2020 Jul 27;12(8):2233. doi: 10.3390/nu12082233 (PMC7469071; doi:10.3390/nu12082233)
Supplement: Supplementary file 1 [file nutrients-12-02233-s001.zip › Fuller et al_Table S2_Antibodies.pdf]

Table S1: Antibody Information

Information describing the primary antibody type, application used in this study and the supplier and supplier catalog number for each antibody

**Primary Antibody**	**Type**	**Application**	**Supplier**	**Catalog Number**
SREBP1	Mouse monoclonal	western blot	Santa Cruz	sc-13551
AKT	Mouse monoclonal	western blot	Cell Signaling	2920
AKT phospho-Ser^{473}	Mouse monoclonal	western blot	Cell Signaling	4051
AMPK	Rabbit polyclonal	western blot	Cell Signaling	2532
AMPK-phospho Thr172	Rabbit polyclonal	western blot	Cell signaling	2535
CD36	Rabbit polyclonal	western blot	GeneTex	GTX112891
PDK4	Rabbit polyclonal	western blot	ABclonal	A13337
SCD1	Rabbit polyclonal	western blot	ABclonal	A16429
β-actin	Mouse monoclonal	western blot	Santa Cruz	sc-47778
