# Supplementary material for: Adaptive Fat Oxidation Is Coupled with Increased Lipid Storage in Adipose Tissue of Female Mice Fed High Dietary Fat and Sucrose
Source: Nutrients. 2020 Jul 27;12(8):2233. doi: 10.3390/nu12082233 (PMC7469071; doi:10.3390/nu12082233)
Supplement: Supplementary file 1 [file nutrients-12-02233-s001.zip › Fuller et al_Table S3_Gene names and Primers.pdf]

Table S2: Gene Name and Primer Information

Information is provided about the gene name and primer design used in this study. All primers were obtained from IDT.

Gene ID	Gene Name	Sequence -Forward	Sequence -Reverse
CD36	CD36 antigen (Cd36)	GGAGTGCTGGATTAGTGGTTAG	TCGTTTCCCACACTCCTTTC
CPT1b	carnitine palmitoyltransferase 1b	CCCGAGCAGTGCCGGGAAGC	GAAATGAGCCAGCTGTAGGG
CPT2	carnitine palmitoyltransferase 2	TGCCCAGGCTGCCTATCCCTAAACT	GCTCCTTCCCAATGCCGTTCTCAAAAT
Cs	citrate synthase	CGGGAGGGCAGCAGTATCGG	ACCACCCTCATGGTCACTATGGATG
PGC1 alpha	peroxisome proliferative activated receptor, gamma, coactivator 1 alpha	AGCCTCTTTGCCCAGATCTTC	CCATCTGTCAGTGCATCAAATGA
PPAR delta	peroxisome proliferator activator receptor delta	CAAGTTCGAGTTTGCTGTCAAG	GTGTCTGGAGTGTTGTGAGTAG
PPAR gamma	peroxisome proliferator activated receptor gamma	CACAATGCCATCAGGTTTGG	GCTGGTCGATATCACTGGAGATC
Chrebp	Carbohydrate Response Element Binding Protein (all variants)	GAAACCTGAGGCTGTCATCCT	CGTGGTATTCGCGCATCA
Elovl6	Elongation of Long Chain Fatty Acids, Family Member 6	CCCGAACTAGGTGACACGAT	TACTCAGCCTTCGTGGCTTT
Fasn	fatty acid synthase	GTCTGGAAAGCTGAAGGATCTC	TCTCGGGATCTCTGCTAAGG
G6pc	Glucose 6-phosphatase	CCTCGTCTTCAAGTGGATTCTG	GGTGACAGGGAACTGCTTTAT
PC	Pyruvate Carboxylase	CCTGCTCGTCAAAGTCATTGC	ACACCTCGGACACGGAACTC
Pck1	phosphoenolpyruvate carboxykinase 1; PEPCK	TATGCTGATCCTGGGCATAAC	TCATGGCCAAGTTAGTCTTCC
SCD1	Stearoyl-Coenzyme A Desaturase 1	GCTGGGCAGGAACTAGTGAG	GGTAGGGAGGATCTGGAAGC
